# Perspectives of Young Women With Gynecologic Cancers on Fertility and Fertility Preservation: A Systematic Review

**DOI:** 10.1093/oncolo/oyab051

**Published:** 2022-02-28

**Authors:** Vânia Gonçalves, Pedro L Ferreira, Mona Saleh, Christina Tamargo, Gwendolyn P Quinn

**Affiliations:** Centre for Health Studies and Research of the University of Coimbra (CEISUC), Faculty of Economics, University of Coimbra, Coimbra, Portugal; Centre for Health Studies and Research of the University of Coimbra (CEISUC), Faculty of Economics, University of Coimbra, Coimbra, Portugal; Department of Obstetrics and Gynecology, Division of Gynecologic Oncology, Icahn School of Medicine at Mount Sinai, New York, NY, USA; Department of Internal Medicine, Johns Hopkins Hospital, Baltimore, MD, USA; Departments of Obstetrics and Gynecology and Population Health, Grossman School of Medicine, New York University, New York, NY, USA

**Keywords:** gynecological cancer, young women, fertility, fertility preservation, attitudes, fertility counseling

## Abstract

**Background:**

Gynecologic cancers standard treatment often requires the removal of some reproductive organs, making fertility preservation a complex challenge. Despite heightened oncofertility awareness, knowledge about fertility attitudes and decisions of young patients with gynecologic cancer is scarce. The aim of this systematic review was to highlight what is currently known about knowledge, attitudes, and decisions about fertility, fertility preservation, and parenthood among these patients.

**Methods:**

Peer-reviewed journals published in English were searched in PubMed, Web of Science and EMBASE from January 1, 2000 to July 1, 2020. Childbearing, fertility, fertility preservation, pregnancy, and parenthood attitudes/decisions after gynecologic cancer from women’s perspective were evaluated.

**Results:**

A total of 13 studies comprised the review. Most of the women valued fertility preservation procedures that could be regarded as a means to restore fertility. A unique feature identified was that fertility preservation was seen also as a way to restore gender identity perceived to be lost or threatened during diagnosis and treatment. Fertility counseling was suboptimal, with wide variability among studies reviewed. Comparisons between gynecologic cancers and other cancer types about fertility counseling rates were inconclusive. The potential negative impact of impaired fertility on patients’ mental health and quality of life was also documented.

**Conclusions:**

Fertility and parenthood were important matters in patients’ lives, with the majority of patients expressing positive attitudes toward future childbearing. Results confirm that the inclusion of patients with gynecologic cancer in research studies focusing on this topic still remains low. Additionally, the provision of fertility counseling and referral by health professionals is still suboptimal.

## Teaser

Standard treatment of gynecologic cancer often requires the removal of reproductive organs, making fertility preservation a major concern. This review summarizes the date on the provision of fertility counseling for women with gynecologic cancers, focusing on their knowledge, attitudes, and decisions related to fertility preservation and parenthood.

**Implications for Practice**

This review recommends that proactive multidisciplinary oncofertility programs that provide optimal fertility counseling should be included routinely in gynecologic cancer care. These programs should be grounded in a robust collaboration among different specialities, with clear pathways for timely referral to fertility specialists. Supplementing these efforts with educational resources, such as Decision Aids, may increase quality of life and patient satisfaction with fertility counseling provided. Timely identification of patients at risk for mental distress and provision of psychosocial interventions are essential to reduce the likelihood of long-term distress. The continuity of oncofertility care should be available to patients at diagnosis and through survivorship.

## Introduction

Gynecologic cancers are a heterogeneous group of malignancies in women, each with different pathological features, clinical presentations and treatment modalities.^1^ However, they share the same threat of potential loss of fertility. Standard treatments involve surgical removal and/or ablative therapies of reproductive tract organs, in addition to adjuvant therapy in the form of pelvic radiation and chemotherapy. These treatments have potential for finite damage to reproductive capacity.^2^ Although gynecologic cancers are more frequent in women over 50 years of age; a subset of younger women are diagnosed during reproductive years. As such, infertility and subfertility can impact this population.

Fertility is a key component of cancer management and quality of life (QOL) in reproductive-aged women. The need for fertility counseling prior to cancer treatments is strongly recommended by clinical guidelines.^3,4^ Fertility preservation provides an opportunity for women to achieve future biological children. Since standard gynecologic cancers’ treatment often requires removal of some reproductive organs, fertility preservation may be a complex challenge for these patients. Personalized counseling by a multidisciplinary team should be advised for each patient,^2,5-7^ including an evaluation of benefits, risks, and safety.^2^ Established fertility preservation options may be considered in addition to fertility-sparing strategies.^6,8-10^ Family building, including egg donation, embryo donation, use of a gestational carrier, and adoption may constitute appropriate alternatives for women who desire to pursue parenthood.^11^

Literature identifying fertility attitudes and decisions of young patients with gynecologic cancer is scarce. While oncofertility reviews have been addressed in the literature for women with breast cancer or cancer in general, there are no reviews assessing young women with gynecologic cancer who have greater risk for fertility loss. It is paramount to understand fertility issues of gynecologic patients and to devise more comprehensive counseling strategies and psychological/educational interventions tailored to their context. The aim of this systematic review was to capture and summarize all relevant data on the incidence of fertility counseling provision for women with gynecologic cancer and their knowledge, attitudes, and decisions related to fertility, fertility preservation, and parenthood from their point of view.

## Materials and Methods

### Search Strategy and Study Selection

**Figure 1.**
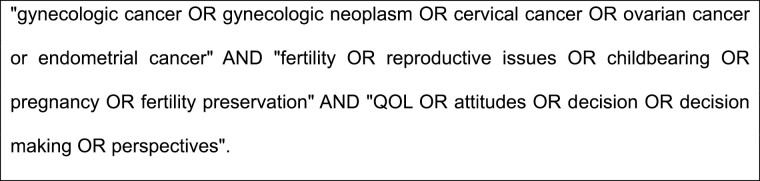
Search string.

A systematic review was conducted using the Preferred Items for Systematic Reviews and Meta-Analysis (PRISMA) guidelines.^12^ Peer-reviewed journals published in English were searched in PubMed from January 1, 2000 to July 1, 2020. The search was conducted using combinations of these phrases or keywords (Fig. 1). An identical search was replicated using Web of Science and EMBASE databases. The following inclusion criteria were applied: (1) studies including young adult patients with gynecologic cancer of childbearing age (when studies used samples of patients with different diagnoses, results needed to be presented specifically for each cancer type) and (2) primary research reporting on the outcomes: childbearing, fertility, fertility preservation, pregnancy, and parenthood attitudes/decisions after gynecologic cancer from patient’s perspective, decisional conflict, and regret regarding fertility decisions. Given the inconsistencies in the literature regarding the definition of young women,^13,14^ we refer to “young” as women 45 years or younger at diagnosis. Review articles, conference abstracts, editorials, commentaries, correspondence, or case reports were excluded. Publications were initially screened based on the title and type of article. Abstracts meeting inclusion criteria were screened for eligibility and the full publications were reviewed. The search was complemented by manually searching the reference lists of included manuscripts. This study was deemed to be nonhuman subjects research by self-certification through the New York University Institutional Review Board.

### Data Analysis and Quality Assessment

Search results were compiled in Covidence (Veritas Health Innovations) and duplicates removed. Titles and abstracts were screened independently by 4 authors (V.G., G.P.Q., C.T., M.S.) to identify eligible manuscripts. Differences were resolved through discussion. The same 4 authors independently reviewed full texts for inclusion in the final sample for extraction. Data extracted from each eligible study were recorded in a standardized extraction table and synthesized using narrative description. To answer the research question, results from original authors’ manuscripts were applied in response to the research questions (what were the knowledge, attitudes, and decisions related to fertility, fertility preservation, and parenthood among young patients with gynecologic cancer?)

The quality of the studies was assessed using the Mixed Methods Appraisal Tool (MMAT), a widely used instrument that allows for quality appraisal of quantitative, qualitative, and mixed methods studies.^15-17^ Each study was rated according to MMAT guidelines.^17^ Since this review intended to capture all published data that met the inclusion criteria, no publications were excluded based on quality ratings. Each study’s quality assessment was performed and discussed by 4 authors (V.G., G.P.Q., C.T., M.S.) (Supplementary Tables S1-S3).

## Results

**Figure 2.**
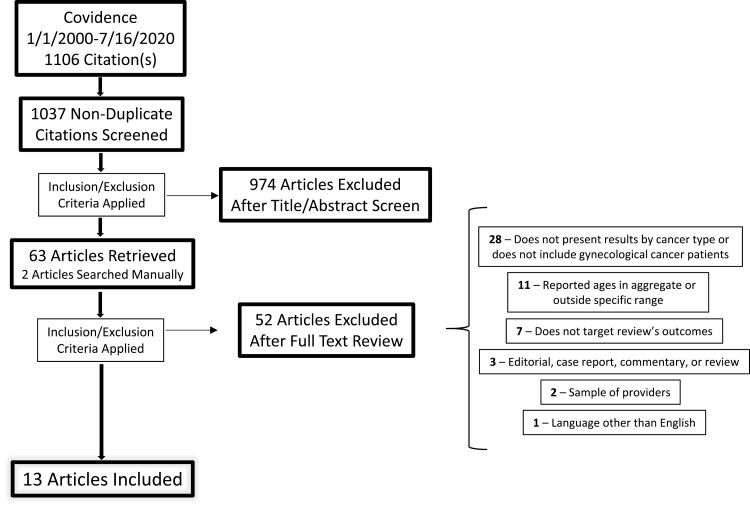
Flow diagram of inclusion/exclusion of papers in the review.

In total, 1106 articles were retrieved; after 69 duplicates were removed, 1037 articles remained (Fig. 2). Of those, 11 studies met the eligibility criteria. Additionally, 2 more articles were obtained manually. In total, 13 studies were included in the review. Table 1 lists all studies included in this review and summarizes the information specific to each study.

**Table 1.** Studies included in the review.

Study	Origin	Type	Aims	Eligibility/inclusion criteria	Sample	Study design	Data collection tool(s)	Relevant findings in GYN cohort	Quality assessment
Wenzel et al^18^	US	Quantitative	To describe the QOL of reproductive-aged women diagnosed with cervical cancer 5-10 years earlier; to identify factors that may compromise or enhance QOL	For cervical cancer survivors: - Cervical cancer survivors diagnosed 5-10 years earlier - 17-45 years of age at DX - No recurrence or second malignancy For controls: - Same race/ethnicity within 5 years of survivors’ age - Not survivors’ blood relatives - No history of hysterectomy - No history of cancer	For cervical cancer survivors: - *N* = 51 - Mean age at DX 37.1 (range 25-45) - Mean time since DX 8 years - 31 (60.8%) married For controls: - *N* = 50 - Mean time since DX: 8 years - 39 (78%) married Differences among groups: - Cases older at interview - Cases had more minorities - Younger cases at interview less likely to be married	Cross-sectional case-control study	SF-36, IES, GPC, SAQ, ISEL, COPE, scale on reproductive concerns designed for this study	- Survivors had more reproductive concerns than controls (*P* < .001) - Reproductive concerns were related to sadness about inability to bear children (31%), inability to talk openly about fertility (30%), frustration related to childbearing inability (25%), and mourning the loss of the ability to have children (25%) - Survivors’ reproductive concerns were associated with poorer QOL (*P* < .0001), more cancer-specific distress (*P* < .01), less social support (*P* < .01), lower spiritual well-being scores (*P* < .05), greater GYN pain (*P* < .0001), and poorer sexual functioning (*P* < .05) - Reproductive concerns were one predictor of individual differences in QOL	3 (∗∗∗), 60%
Wenzel et al^19^	US, UK	Quantitative	To assess psychosocial and reproductive concerns and QOL in long-term female cancer survivors and assess the relationship between infertility and long-term QOL	For GYN cohort: - Women diagnosed with/treated for cervical cancer during childbearing age 5-10 years earlier - 17-45 years of age - No recurrence or second malignancy Other groups: - Lymphoma survivors - GTT survivors - Acquaintance controls (unmatched)	Cervical cancer cohort: - *N* = 51 - Mean age at Dx 37 - Mean time since DX 8 years - 31 (60.1%) married Lymphoma cohort: - *N* = 69 - Mean age at DX 32.7 - Mean time since DX 7.2 years - 54 (78.2%) married GTT cohort: - *N* = 111 - Mean age at DX 29.8 - Time since DX 7.2 years - 90 (81.8%) married Controls: - *N* = 148 - Mean age at interview 40 - 106 (72.6%) married Existence of children prior to dx not stated	Cross-sectional	SF-36, QRTL-CS, IES, ISEL, GPC, COPE, self-reported infertility measured by 4 questions, scale on reproductive concerns designed for this study	- For general sample, having more reproductive concerns was associated with poorer physical/mental health (*P* < .001), greater GYN problems (*P* < .01), more cancer-related distress (*P* < .001), and less social support (*P* < .001) - Social support, GYN problems, and reproductive concerns significantly predicted individual differences in overall QOL, controlling for age at DX/type of DX; less social support, more GYN problems, and more reproductive concerns had significantly worse overall QOL - Infertile women had worse mental health, more cancer-specific distress, and lower overall/physical/psychological well-being - Cancer survivors had more reproductive concerns than controls; cervical cancer survivors had more concerns than other survivors, but the difference was not significant	4 (∗∗∗∗), 80%
Carter et al^20^	US	Quantitative	To assess emotional and sexual functioning, reproductive concerns, and QOL in women with cancer-related infertility compared to those without a cancer history; to explore awareness of third-party reproduction options in cancer survivors	For cancer survivors: - History of GYN cancer or history of leukemia, lymphoma, or sarcoma status post BMT/SCT - No evidence of disease for at least 1 year - No other cancer hx - 18-49 years of age at recruitment - Have not started or have not completed childbearing - English-speaking For non-cancer infertile women: - No cancer history - 18-49 years of age at recruitment - History of infertility and on waitlist for oocyte donation - Have not started or have not completed childbearing - English-speaking	For GYN cancer sample: - *N* = 51 - 43 (84%) cervical, 1 (2%) ovarian, 3 (6%) endometrial/ uterine, 4 (8%) GTT - Mean age at DX: 34.8 - Mean length of time since dx: 3.8 - 37 (73%) married or cohabitating - Existence of children not stated	Cross-sectional	RCS, CES-D, FSFI, IES, Menopausal Symptom Checklist, SF-12 Health Survey, ADAS	- 12 (24%) agreed that “fertility played a factor in your decision about cancer TX” - 35 (69%) reported inadequate time to complete childbearing - No difference about reproductive concerns between groups, although mean scores elevated compared to published values - Rated importance of parenthood 8.80 (0 = not at all, 10 = extremely important); 36 (71%) gave a value of 10 compared to 48% BMT/SCT survivors - 24 (47%) worried about how a cancer DX and TX may affect their offspring - 28 (55%) did not feel they had fertility options, compared to 25 (35%) in BMT/SCT group (*P* = .023) - 25 (49%) knew where to go or with whom to speak regarding reproductive assistance, compared to 52 (73%) of the BMT/SCT group (*P* = .013) - 32 (63%) indicated it would be/was helpful to speak with a fertility counselor or reproductive specialist; 17 (33%) had spoken to one - 9 (18%) had used assisted reproductive techniques - 50 (98%) were familiar with surrogacy and 27 (53%) viewed it as a viable option - 36 (71.5%) viewed alternatives to childbirth like adoption or foster parenting as acceptable - 18 (35%) were concerned about trying to adopt as a cancer survivor - 37 (72.5%) had heard of oocyte retrieval and 18 (31%) had considered it - 38 (74.5%) had heard of oocyte donation and 31 (61%) had considered it - The order of the most acceptable reproductive techniques was adoption (24, 47%), surrogacy (16, 31%), egg donation (9, 18%), and fostering (2, 4%)	4 (∗∗∗∗), 80%
Armuand et al^21^	Sweden	Quantitative	To investigate male and female cancer survivors’ perceptions of fertility-related information and use of FP in connection with cancer TX during reproductive age	- Diagnosed with lymphoma, acute leukemia, testicular cancer, or ovarian cancer at 18-45 years of age, or female breast cancer survivors treated with chemotherapy	For ovarian cancer sample: - 17/484 patients - Mean age at DX: 34.7 - 12 (70.6%) married or cohabitating - 9 (52.9%) with children at dx - Length of time since DX not stated	Cross-sectional	Study-specific questionnaire about fertility-related information and use of FP	- 5 (29.4%) received information about TX impact on fertility - 4 (23.5%) received information about FP (3 about OC, 1 about OTC) - None had FP	3 (∗∗∗), 60%
Campos et al^22^	US	Quantitative	To increase knowledge about the needs of patients with ovarian cancer who underwent FSS; to assess the feasibility of this study	- Diagnosed with early-stage ovarian cancer or borderline ovarian malignancy - <40 years of age - Underwent FSS - Completed therapy for early-stage cancer - Without evidence of recurrence - Without prior DX of infertility or significant medical condition that would affect fertility	- *N* = 16 - Median age at DX: 30 (range 22-39) - Median age at study: 31.5 (range 23-41) - 69% *<*2 years since DX - 25% married - 75% never pregnant	Cross-sectional	Ovarian Cancer or Borderline Malignancy of the Ovary: Fertility Sparing Survey (devised by study author, not validated), Sexual Activity Questionnaire	- 100% of patients intended become pregnant in the future - 88% considered FSS very/extremely important - 87.5% discussed fertility-sparing surgery as an option - 3/16 (20%) were counseled by a fertility specialist - 12 (75%) were advised to consider chemotherapy - 11/12 (91%) had fertility counseling with their clinicians - 4 (33%) advised to pursue chemotherapy were counseled by a fertility specialist; 10 of those (84%) did not use FP measures	3 (∗∗∗), 60%
Chan et al^23^	US	Quantitative	To compare regret in GYN cancer survivors who did and did not recall pre-TX fertility counseling; secondary aim to evaluate the effect of FSS on regret and to characterize patients at highest risk of regret	- History of localized (stage 1) cervical, ovarian, or endometrial cancer - 18-40 years of age at DX - Dx between 1993-2007	- *N* = 470 - 228 (48.5%) cervical, 125 (26.6%) ovarian, 117 (24.9%) endometrial - Mean age at dx: 33.7 - Mean age at survey: 45.2 - 324 (69%) had children before TX - 235 (50%) desired children after TX	Cross-sectional	DRS; additional measures on demographic, health, reproductive health information, QOL, decisional satisfaction and regret, and existence and satisfaction with fertility counseling	- 206 (46%) recalled pre-TX fertility counseling from an oncologist/surgeon, 47% of whom were satisfied with the counseling - 182 (39%) underwent FSS - There was no association between FSS and regret - After adjusting for age at time of DX and at time of survey, counseling (*P* = .02) and FSS (*P = .*03) were associated with lower regret scores - Desire for more children at time of DX was associated with higher regret (*P* < .001 adjusted)	4 (∗∗∗∗), 80%
Ameri et al^24^	Iran	Quantitative	To evaluate awareness of fertility impairment following TX in female cancer patients of childbearing age	- Female - Diagnosed with any type of cancer - Did not have any severe medical condition or permanent infertility before DX	- 9/247 (3.6%) cervical cancer, 17/247 (6.9%) ovarian cancer - Ages (not published): 18-44 ovarian, 36-44 cervical - Marital status and existence of children at DX not stated by cancer site	Cross-sectional	Study-specific questionnaire about the risk of infertility following cancer TX	- 56/247 (22.7%) received fertility information - 7/9 cervical and 9/17 ovarian patients (51.6% of GYN cancer patients) received information/ counseling about infertility, compared to 40 (18.5%) with other cancers (*P* < .0001) - 80% of GYN patients receiving pelvic radiation received information about fertility, compared to 25% of pelvic radiation patients with other cancers	3 (∗∗∗), 60%
Sobota et al^25^	UK, Poland	Quantitative	To assess the determinants of fertility-related distress with a cross-cultural perspective using the CSM	- Diagnosed with GYN or breast cancer - 18-45 years of age at DX - Menstrual at time of DX - Received chemotherapy (if breast cancer) - Completed active TX - No known evidence of cancer recurrence - English- or Polish-speaking	For GYN cancer sample: - *N* = 129 (78.7%) - 58 (45.0%) cervical, 41 (31.8%) ovarian, 27 (20.9%) uterine, 3 (2.3%) other GYN For total sample: - *N* = 164 - 118 (72.0%) British, 43 (26.2%) Polish, 2 (1.2%) other, 1 (0.6%) missing - 80 (48.8%) had no children before treatment - Data on these variables specific to GYN cancers not listed	Cross-sectional	IES-R; Brief-IPQ; negative affect subscale PANAS; VOC scale; single items designed for this study measuring decisional regret, social disapproval of not having children, and one’s own and partner’s desire to have children at time of cancer DX	- Fertility-related distress was not predicted by type of DX - Determinants of distress were country of origin, recruitment site, negative affect, desire to have children, treatment regret, and total illness perception score - The impact of the desire to have children on fertility-related distress was mediated by the psychological value of children, perceived consequences of cancer on one’s life, emotional representation, and treatment-related regret - Country of origin moderated the relationship between the desire to have children and fertility-related distress when mediated by treatment-related regret	3 (∗∗∗), 60%
Shah et al^26^	US	Quantitative	To explore patients’ perceptions of preoperative reproductive counseling; to evaluate postoperativeerative complications and pregnancy outcomes in patients who underwent radical trachelectomy for early-stage cervical cancer	- Women with cervical cancer - Underwent radical trachelectomy between January 01, 2004-July 31, 2017 - Were cancer-free >1 year after radical trachelectomy	- *N* = 39 - Median age at dx 37 (range 25-37) - 12 (31%) had children before dx - Length of time since dx and marital status not stated	Cross-sectional	Study-specific questionnaire with quantitative and qualitative items	- 18 (46%) had reproductive counseling prior to radical trachelectomy; 14/18 (78%) had counseling from a GYN oncologist, 7 (39%) from a reproductive endocrinologist, 1 (6%) from a maternal-fetal specialist, and 1 (6%) from a women’s health nurse; 4 patients received counseling from at least 2 providers; 16 (89%) found counseling adequate and helpful in making an informed decision - 29 (68%) received counseling about pregnancy risks and complications; 23/26 (88%) found it adequate and 22 (85%) found it helpful in making an informed decision; 25 (96%) were counseled on these matters only by GYN oncology - Median level of anxiety was similar before and after TR - Desire for pregnancy was lower 6 months after surgery than it was at time of radical trachelectomy (*P = .*02) - 90% would still choose radical trachelectomy over radical hysterectomy if making the decision again - Preoperative counseling was seen as important - radical trachelectomy was viewed as having a positive impact on patients’ lives	3 (∗∗∗), 60%
Chin et al^27^	US	Quantitative	To assess which characteristics are associated with failure to receive fertility counseling among a cohort of young women diagnosed with cancer	- 20-35 years of age at DX with invasive cancer or DCIS during 1990-2009 - 22-45 years of age and *>*2 years after DX at recruitment - With working telephone - English-speaking - No history of hysterectomy or bilateral salpingo-oophorectomy before DX	- 67/1116 (6.0%) reproductive (cervical, ovarian, uterine) cancer - Mean age at DX, length of time since DX, marital status, and existence of children not stated by cancer site	Population-based cohort	Telephone interview piloted by authors and reported in previous study	- 51/63 (81%) received fertility counseling - GYN cancer survivors were the most likely group to have been counseled	3 (∗∗∗), 60%
Komatsu et al^28^	Japan	Qualitative	To explore the experience of FP with radical trachelectomy from the perspective of women with cervical cancer	- Women with cervical cancer - Underwent radical trachelectomy between 2006 and 2010 - Were followed at an outpatient clinic and encouraged to attempt conception after 6 months without cancer recurrence	- *N* = 15 - Mean age at time of surgery 31.6 years (range 25-38) - 6/15 (40%) were married at time of surgery - 1/15 (6.7%) had children before surgery	Qualitative interviews	Semi-structured interviews	- Feminine identity is first threatened by cancer and then repaired by FP in patients who underwent radical trachelectomy - Feminine identity is reconstructed after radical trachelectomy through interactions with self, others, and external events - Loss of uterus contributed significantly to women feeling their feminine identity was damaged - FP was significant - The meaning of FP varied - Childbearing was not the only expected outcome of FP	5 (∗∗∗∗∗), 100%
Mitchell et al^29^	US	Qualitative	To determine patients’ knowledge and feelings about their OTC	- *>*18 years of age - Underwent OTC between 2006 and 2017 at a single academic center	- 3/8 GYN cancer - Mean age 25.8 years - Time since DX and existence of children not reported	Qualitative interviews	Interview guide created by authors (details not reported)	- Participants had positive feelings about having done OTC and desired future fertility - There was lack of knowledge regarding OTC - There is a need for more education and follow-up care from healthcare providers	4 (∗∗∗∗), 80%
Carter et al^30^	US	Mixed methods	To prospectively assess and describe the emotional, sexual, and QOL concerns of women with early-stage cervical cancer undergoing radical surgery	- 18-45 years of age - Diagnosed with early-stage cervical cancer - Consented for radical trachelectomy or radical hysterectomy - No history of chemotherapy or radiation	For pre-surgery sample: - *N* = 71 - Mean age at DX: 34.5 (range 20-45) - 41 (58%) married or cohabitating - 23 (33%) had children at enrollment - 43 (61%) had radical trachelectomy; 28 (39%) had radical hysterectomy Post-surgery sample: - *N* = 52 completed at least 2 time points	Prospective cohort (time points: pre-surgery; 3, 6, 12, 18, and 24 months post-surgery)	FACT-Cx, CES-D, IES, FSFI. Qualitative items focusing on fertility issues	- For radical trachelectomy group, fertility and not enough time to complete childbearing were factors in the TX decision-making process for most women; 42 (97.7%) desired ovarian preservation for future fertility or for menopause prevention; fertility (23, 55%), doctor discussion/recommendation (15, 36%), and research (7, 17%) were important factors guiding TX choice - 3 (7%) of radical trachelectomy group had enough time to complete childbearing compared with 12 (43%) of radical hysterectomy group (*P* < .001) - For radical hysterectomy group, fertility and childbearing were factors in tx choice for about half of women (46%); 6 (21.4%) desired ovarian removal	2 (∗∗), 40%
								- Reasons for choosing this surgical procedure included themes of doctor discussion/recommendation (11, 46%), “concern about survival” (6, 25%), and feeling this was the “best option or only choice” (6, 25%) - In radical trachelectomy group: 39 (91%) pre-surgery, 29/33 (88%) at 6 months, 25/33 (76%) at year 1, and 24/33 (73%) at year 2 had concerns about trying to conceive in the future - When asked, “How successful do you think you will be at conceiving in the future,” mean ratings were 61.8% pre-surgery, 53.7% at 6 months, 55.4% at year 1, and 59.8% at year 2 - 5 (15%) spoke to an infertility specialist by year 1 and 6 by year 2 - At year 1, 2 (6%) were trying to conceive, 3 (9%) had achieved conception, and 2 (6%) were pregnant - At year 2, 7 (21%) were attempting conception, 5 (15%) had conceived, and 3 (9%) were pregnant - At completion, 1 patient was pregnant and 8 babies had been born - Type of surgery was not related to measures of mood, distress, sexual function, or QOL; prospective data failed to show significant differences among radical trachelectomy and RH, highlighting an adaptive process over 2 years	

ADAS, Abbreviated Dyadic Adjustment Scale; BMT/SCT, bone marrow transplant/stem cell transplant; Brief-IPQ, Brief Illness Perception Questionnaire; CES-D, Center for Epidemiologic Studies Depression Scale; COPE, Coping Orientations to Problems Experienced; CSM, Common Sense Model; DCIS; ductal carcinoma in situ; DRS, Decisional Regret Scale; DX, cancer diagnosis; FACT-Cx, Functional Assessment of Cancer Therapy; FP, fertility preservation; FSFI, Female Sexual Function Index; FSS, fertility-sparing surgery; GPC, Gynecologic Problems Checklist; GTT, gestational trophoblastic tumor; GYN, gynecologic cancer; IES, Impact of Events Scale; IES-R, Impact of Event Scale Revised; ISEL, Interpersonal Support Evaluation; OC, oocyte cryopreservation; OTC, ovarian tissue cryopreservation; PANAS, Positive and Negative Affect Scale; QOL, quality of life; QOL-CS, Quality of Life Cancer Survivorship; RCS, Reproductive Concerns Scale; SAQ, Sexual Activity Questionnaire; SF-12, Medical Outcomes SF-12 Health Survey; SF-36, Medical Outcomes Study Short Form 36-item; TX, cancer treatment; VOC, Value of Children Scale.

The majority of studies were quantitative (*n* = 10). Of those, 9 were cross-sectional^18-26^ and one was a population cohort.^27^ The studies were of reasonable quality, ranging from 60% to 80% on the MMAT tool. Five studies used heterogeneous samples of different cancer diagnoses. In addition, 5 studies used samples of patients with gynecologic cancer exclusively. Three studies included only cervical cancer,^18,19,26^ of those, 2 used the same sample;^18,19^ one study used only patients with ovarian cancer;^22^ and one study used a mixed sample of different gynecologic cancer diagnoses.^23^ In total, 910 patients with gynecologic cancers participated in the studies. Of those, 428 had cervical cancer, 217 had ovarian cancer, 147 had endometrial/uterine cancer, and 118 had another gynecologic cancer diagnosis. In the study by Chin et al,^27^ 63 patients of different gynecologic diagnoses (cervical, ovarian, and uterine cancers) participated; however, the authors did not provide numbers of patients by diagnosis. Across all studies, the mean age at diagnosis ranged from 23 to 41 years. Three studies failed to report this information specific to each cancer site.^24,25,27^ Length of time since diagnosis varied among studies, ranging from 3.76 years to 11.6 years. Campos et al^22^ solely stated that 69% of participants were diagnosed within 2 years or less and 31% were diagnosed more than 2 years. In addition, 2 studies did not provide this information by cancer site^21,27^ and 2 studies failed to report information on this variable.^24,25^

Two qualitative studies, using interviews to collect data, explored women’s views on fertility and fertility preservation surgery and cryopreservation of ovarian tissue, respectively.^28,29^ In total, 18 patients participated in those studies. Participants’ mean age at diagnosis/surgery ranged between 25.8 and 31.6 years. These studies were of good to very good quality, ranging from 80% to 100% on the MMAT tool.

Only one study used mixed methods.^30^ A prospective cohort design reporting longitudinal data with a sample of patients with early-stage cervical cancer (*n* = 71) compared, over 2 years at different time points, patients who underwent radical trachelectomy with patients who had radical hysterectomy. Participants were, on average, 34.54 years at diagnosis. Data were collected by self-reported questionnaires, containing qualitative items exploring fertility and other issues. This study had low quality with a score of 40% on the MMAT.

### Fertility Counseling

Among 6 studies reporting fertility counseling provision, a wide range of rates were observed, from 29.4% to 91%.^21-25,27^ Fertility counseling satisfaction was also evaluated by 2 studies. Chan et al,^23^ assessed 470 women, of those 206 (46%) recalled pre-treatment fertility counseling from their oncologist or surgeon; of those, 47% reported satisfactory fertility counseling. Conversely, Shah et al^26^ reported that among the 18/39 patients (46%) who received counseling prior to RT, 16/18 (89%) believed it was adequate and helped to make informed decisions. Furthermore, counseling regarding pregnancy risks and complications before RT was reported among 26/38 patients (68%), of whom 88% stated counseling was appropriate and 85% reported it helped to make informed decisions.^26^ The majority of fertility discussions were conducted before cancer treatment,^21,22,26,27^ predominantly received in person with a minority of women receiving written information^21^ and initiated by the oncologist,^24,26,27^ the patient or the partner.^27^ However, with the exception of Shah et al,^26^ data on these communication issues were not provided by patients but by the entire sample with different diagnoses. Chan et al^23^ also reported patients received counseling from surgeons, and other sources (eg, gynecologists, medical oncologists, general practitioners, and nurses) and from more than one provider.^23^ A similar pattern was observed in patients with cervical cancer who received counseling mainly from their oncologists but also from other sources (eg, reproductive endocrinologist, maternal fetal medicine specialist, and nurse) with some patients receiving counseling from more than one provider.^26^ Among all counseled patients in the Chin et al ^27^ study (*n* = 660), only 12.9% were referred to a fertility specialist. Low rates of referral to fertility specialists were also observed in 3 studies conducted exclusively with gynecologic cancers.^22,26,30^ In this context, Carter et al^30^ examined patients who spoke to a fertility specialist at 2 points in time. Results showed 15% (*n* = 5/33) had a consultation in the first year and, this increased by year 2 (*n* = 6/33). Campos et al^22^ found 20% (3/16) were counseled by fertility specialists’ presurgery. A slightly higher rate was captured by Carter et al,^20^ with a subset of 33% (17/51) patients reporting they had spoken to a fertility specialist, and Shah et al,^26^ who showed that among the 18 patients who received counseling (in a sample of 39 patients), 39% were counseled by a reproductive endocrinologist.

Across different cancer types, women with gynecologic cancers were more likely to report receiving fertility counseling.^24,27^ Accordingly, Ameri et al^24^ compared women who received pelvic radiotherapy, reporting 80% of patients with gynecologic cancer recalled fertility discussions while 25% of women with other diagnoses recalled those discussions. However, this finding was not consistent in another study,^20^ where gynecologic cancer survivors expressed significantly more fertility-related informational needs than other survivors, such as Bone Marrow/Stem Cell Transplant (BMT/SCT).

### Attitudes and Decisions Toward Fertility, Parenthood, and Fertility Preservation

Women expressed positive attitudes toward fertility and parenthood,^20,22,26,28,29^ while concerns or fears about pregnancy and having children after cancer^20,28^ were also reported. Carter et al^20^ found that fertility issues influenced cancer treatment decisions for one-quarter of survivors (*N* = 51), while the majority stated inadequate time to complete their childbearing plans. For example, Campos et al^22^ reported 100% of participants had intention to become pregnant in the future. Carter et al^20^ reported that overall, parenthood was perceived as the highest importance in life by most survivors; however, almost half also worried about the diagnosis and treatment effect on their offspring’s’ health. This worry was also voiced by cervical cancer survivors who underwent RT, with reports of women preferring childless life after surgery.

Complications associated with fertility procedures may determine negative views toward fertility preservation. Shah et al^26^ reported 10% of participants (N-4) who underwent radical trachelectomy expressed that if they had to make the decision again, they would choose radical hysterectomy instead of radical trachelectomy namely due to physical symptoms (*n* = 3) and psychological distress given multiple failed pregnancies (*n* = 1). Cancer recurrence concerns also equated to avoidance of sexual activity and attempting conception for some.^28^

Women expressed perspectives on fertility preservation decisions, such as radical trachelectomy for early-stage cervical cancer,^26,28,30^ cryopreservation of ovarian tissue,^29^ and family building.^20^ The qualitative study by Komatsu et al,^28^ captured the “meaning” women attributed to their reproductive organs, which were perceived as the core of gender identity, parenthood, sexuality, and female identity. The use of radical trachelectomy provided an opportunity to conceive in the future and a means to restore threatened gender identity since the potential loss of their uterus brought feelings of being incomplete. Participants reported high satisfaction with decision making, even if reproductive outcomes were not achieved. This perception was emphasized by some reporting feeling pressured by health providers to pursue childbearing because they underwent a fertility preservation surgery. The authors highlighted the cultural context where the study took place in which social norms and views of family are often influenced by feminine identify. Positive views toward radical trachelectomy were described by Shah et al^26^; 90% of participants reported they still would choose radical trachelectomy over radical hysterectomy again. Responses to open-ended questions showed gratitude for the possibility to undergo radical trachelectomy and the procedure had a positive impact on their lives. Carter et al^30^ also identified motives leading to the choice of this fertility preservation surgery instead of radical hysterectomy. The desire to preserve fertility was a decision factor for the majority of women (42/43). In the radical trachelectomy group, women expressed not having had enough time to complete childbearing compared to radical hysterectomy group. Physician recommendations and, to a lesser extent, research were other reasons to pursue radical trachelectomy. About half of radical hysterectomy group (13/28; 46%) cited fertility as a factor in their treatment choice. In qualitative responses, the radical hysterectomy group referred to physician recommendation, and recurrence, to guide their decisions. Concerns about future conception were examined prospectively in the radical trachelectomy group, showing that at presurgery, 39 patients (91%) had concerns. The numbers of patients with concerns decreased to 24/33 (73%) by Year 2. Women’s perceptions of future successful conception showed presurgery ratings of 61.8%, and at 6 months 53.7%, followed by 55.4% at year 1, and at year 2 was 59.8%.^30^ Positive views toward fertility-sparing surgery were also expressed by patients with ovarian cancer with the majority of participants (88%) expressing the surgery was important to them.^22^

A desire for future fertility and positive feelings about ovarian tissue cryopreservation, were also expressed in a qualitative study among women diagnosed with gynecologic cancer who underwent this fertility preservation procedure.^29^ Quotations from these women highlighted a lack of knowledge about the procedure and a need for improved patient education and follow-up care from health providers regarding the process.

Perceptions of lack of availability of fertility preservation options were expressed by gynecologic cancer survivors (55%; 28/51) compared to BMT/SCT survivors (35%; 25/71).^20^ Most of gynecologic cancer survivors were familiar with surrogacy parenting options, such as surrogacy, and had heard of oocyte retrieval and oocyte donation. Oocyte retrieval was considered by 31% (16/51) of gynecologic cancer survivors, while 61% (31/51) considered oocyte donation and 53% (27/51) perceived surrogacy as a viable option. Although concerns about adoption as a survivor were raised by 35% (18/51), the majority (36/51; 76%) expressed it would be acceptable to explore alternatives such as adoption or fostering a child. Adoption was perceived as the most acceptable alternative, followed by surrogacy, egg donation, and fostering.^20^

### Fertility-Related Psychosocial Impact

Some authors focused on the impact of fertility issues on psychosocial outcomes.^18,19,25,30^ Data analysis of patients’ questionnaires showed that reproductive concerns were associated with poorer QOL, more cancer-specific distress, less social support, lower spiritual well-being, greater gynecologic pain, and poorer sexual functioning in 51 cervical cancer survivors.^18^ In addition, questionnaires’ data also revealed that reproductive concerns were related to sadness about inability to bear children (31%), inability to talk openly about fertility (30%), frustration related to childbearing inability (25%), and mourning the loss of ability to have children (25%). Compared to controls, cervical cancer survivors expressed significantly more reproductive concerns.^18,19^ However, when compared with survivors of lymphoma or Gestational Trophoblastic Disease, differences were not significant.^19^ Reproductive concerns’ increase was influenced by feelings of anger and grief related to loss of reproductive ability.^19^ Similarly, Carter et al^30^ found an increase in reproductive concerns among gynecologic cancer survivors, BMC/STC survivors, and infertile women without cancer, compared with normative data; however, there were no significant differences among groups. Reproductive concerns were one of the predictors of individual differences in QOL in patients with cervical cancer^18^.

Fertility counseling and patients’ satisfaction were associated with reduced fertility decisions’ regret.^23^ Fertility-sparing surgery also yielded reduced decisional regret.^23^ Sobota et al^25^ reported on the relationship between regret and the impact of cancer treatments on fertility and the increased risk of fertility-related distress. Findings suggested that treatment-related regret mediated the relationship between desire to have children and fertility-related distress. This mediation analyses were further explored regarding socio-cultural influences on regret showing that country of origin acted as a moderator of this relation. Therefore, for British participants, a greater desire to have children contributed to increased treatment outcome regret and indirectly to more fertility-related distress. For Polish women, the desire to have children was related to distress, but did not have effect on regret. Authors speculated the medical system differences between the 2 countries and women’s awareness of fertility preservation opportunities may have been reflected in those results.

A desire for children at diagnosis predicted fertility distress post-treatment^25^ and it was associated with increased regret.^23^ The inability to fulfill this desire was associated with more reproductive concerns and greater need for coping efforts.^19^ Sobota et al^25^ studied the relationship between desire for children and fertility-related distress, reporting the psychological value of children, perceived consequences of cancer, emotional burden, and treatment regret mediated this relationship. Wenzel et al^19^ showed that self-reported infertile women were significantly more likely to express poorer mental health, more cancer-specific distress, reduced overall, physical and psychological well-being that those who did not report fertility problems.

Illness perceptions, namely the emotional representations (expressed in more concerns about the illness and more emotional burden about the cancer diagnosis) rather than the cognitive representations, negative affect, recruitment site (patients’ recruited through clinics rather than online), and country of origin (Poland vs. UK) were also predictors of fertility distress.^25^ The later predictor reflected the finding that Polish participants were more likely to experience higher fertility distress. Authors attributed this result to possible cultural differences between Poland and UK regarding family values often attached to religious beliefs.

Women’s perceived knowledge of family building options was associated with depression and distress levels, in a way that women with a perceived need for more information showed significantly more depression and avoidance than women who expressed no informational needs among gynecologic and BMT/STC survivors.^20^ No differences on mood, reproductive concerns, and mental health QOL among this group of cancer survivors and infertile non-cancer women were found. According to the authors, the emotional experience of infertility is similar among women, regardless of the root of infertility, rather than the premise that cancer survivors experience a double-trauma response of having both cancer and infertility compared to women with non-cancer-related infertility.^20^

## Discussion

Despite guidelines^3,4^ advocating the vital need to routinely include fertility counseling in cancer management and the growing body of research addressing fertility for reproductive-aged patients, this systematic review confirms the inclusion of patients with gynecologic cancer in research focusing on this topic remains unacceptably low.

The provision of fertility counseling and fertility specialists’ referral by healthcare professionals is still suboptimal. Nonetheless, collectively, the study findings corroborate the universal view that fertility is an important matter in young patients’ lives, and a crucial issue during diagnosis and survivorship.^31-34^ One primary aspect that emerged from this review is the wide variability of fertility counseling rates, which, may be a reflection of studies’ different methodological approaches and shared decision making oncofertility practices among individual clinicians or hospitals. This substantial variability was also described by other systematic reviews reporting on fertility counseling for young patients living with cancer with different diagnosis.^31,33^ Comparisons of fertility counseling rates among gynecologic cancers and other diagnosis yielded conflicting results. Two studies in this review documented that patients with gynecologic cancer were more likely to receive fertility counseling^24,27^ due possibility to the cancer being located in the reproductive organs and to the greater awareness of gynecologists or gynecologic oncologists about fertility counseling due to their training.^24,34^ However, this view was disputed by other studies,^20,21^ which corroborated findings suggesting that patients with gynecologic cancers were less likely to have fertility discussions and therefore opportunities for preservation.^35,36^ Across all studies, there is the finding that a significant proportion of patients with gynecologic cancer did not receive fertility counseling or were not referred to fertility specialists. The lack or insufficient fertility counseling occurred even among women who underwent fertility preservation procedures, with some reporting that they underwent these procedures with little knowledge of the implications, risks, and possible reproductive outcomes. Many women in the studies expressed desire for more fertility support^26,29^. Fertility preservation among patients with gynecologic cancer poses unique challenges.^11^ It appears preservation methods are offered to selected patients based, for example, on the type and stage of malignancy.^5^ A multidisciplinary collaboration between gynecologic oncologists and fertility specialists, prior to initiation of cancer treatments, is imperative to determine the suitability of fertility-sparing treatments, assessing reproductive potential, setting realist expectations regarding future pregnancy, and optimizing opportunities for counseling.^37^

From a psychological point of view, a collaborative care model between oncology and fertility specialists has been linked to improved patient knowledge and understanding, better decision making, and reduced long-term regret and dissatisfaction with fertility preservation.^38^ This review supports the optimization of the collaborative model of care to guide oncofertility management for patients with gynecologic cancer as well as a means to improve low referral rates to fertility specialists.^23,26,27,30^ Such models may improve women’s perceived low fertility counseling satisfaction.^23^ In this review, patients of reproductive age prioritize information provision.^39^ Educational tools, such as written information has been reported by patients as a valuable resource.^40^ Unfortunately, the topic of educational information provision was scarcely explored, with only one study reporting on low availability of written information.^21^ Educational instruments, such as Decision Aids (DAs) are useful to facilitate the decision-making process, especially when tailored to patients’ needs.^41^ To our knowledge, there are no validated DAs specifically for gynecologic cancers. However, there is a validated DA for all cancer types, in German^42^ and a Dutch cancer-specific DAs tailored to cancer type, including cervical, endometrial, and ovarian cancers, which is under development.^43^

Understandably, there are ethical issues associated with fertility preservation among gynecologic cancers especially those with metastatic disease. While clinical guidelines suggest fertility counseling be offered to all women, regardless of disease type or stage, many clinicians are hesitant to discuss preservation with patients who have a poor chance of survival.^44^ Further, as some patients may require hysterectomy, carrying a future pregnancy would not be an option and would require the use of a gestational carrier, a practice which is financially unaffordable for some and illegal in several countries.^45,46^

Fertility and parenthood were generally viewed positively by the majority of patients,^20,26,28,29,30^ with the desire to preserve fertility being one of the reasons guiding treatment decisions, such as radical trachelectomy instead of radical hysterectomy in patients with cervical cancer.^20,26,28^ However, pursuing childbearing also trigged patients’ worries namely related to future offspring health and cancer recurrence fears.^20,21,28^ These concerns have also been shown to lead to avoidance of sexual intimacy,^47^ fear of pregnancy or preference for more radical surgical procedures. A unique feature to gynecologic cancers identified in this review was that retaining the ability of childbearing was a means to restore a woman’s sense of gender identity perceived to be lost or threatened during diagnosis and treatments.^28^ In this regard, reproductive organs were seen as the core to parenthood, gender identity, and sexuality. To our knowledge, there are no reports of this type of gender identity perception in relation to reproductive organs in other cancer populations. These perceptions were often fuelled by cultural and family beliefs and norms that may shape women’s attitudes and future decisions regarding the choice of fertility preservation procedures.^28^ This awareness by health professionals of the impact of cancer on women’s own perceptions and attitudes is crucial when providing fertility counseling to patients with gynecologic cancer. The ability to address women’s psychosocial needs and support them in meaningful ways, including addressing their partners or support people is key to patient-physician quality communication.^48^ For example, findings from this review showed that some women felt pressured by their health professionals to attempt a pregnancy, because they had chosen fertility-sparing surgery.^28^ patients with gynecologic cancer held positive attitudes toward alternative family building, with adoption being the most acceptable option, in spite of known reports of discrimination in domestic and international adoption related to a history of cancer.^49,50^ Since fertility preservation in patients with gynecologic cancer may be challenging, family building may constitute a viable option to pursuing parenthood. More research is needed regarding women’s views and attitudes on this topic taking into consideration the psychosocial implications^51^ and the remarkable asymmetries in cultural norms, legislation, and accessibility that exist worldwide regarding family building options.^52^

Some factors identified in this review related to poor mental health are modifiable, such as providing timely and comprehensive fertility counseling, which may have, among other psychological outcomes, a positive impact on future regret regarding fertility decisions.^23^ Conversely, other factors may be inevitable, such as the desire for children at diagnosis with the potential for infertility due to cancer treatments. For those, psycho/logical support, should be provided to support patients within the immediate impact of diagnosis and complex fertility decision-making process and at survivorship, bearing in mind that fertility attitudes and decisions and psychosocial adjustment may evolve over time.^30,53^

A noteworthy aspect raised by Sobota et al^25^ was the cross-cultural differences regarding fertility attitudes, perceptions, and fertility preservation options available after the diagnosis across different countries, which may have varied implications for patients’ mental health and decisional process. Attention to culture and mores is important when devising effective interventions, international guidelines, and counseling models. Larger and rigorous studies are needed to inform comparisons among groups and assess the role of cultural beliefs in oncofertility models. In practice, with the current evidence, health professionals need to be aware of the impact of cultural beliefs and norms regarding fertility when dealing with emigrants, minorities, or ethnical groups, to provide specific support and information they require.^54^

There are some limitations in this review. First, only peer-reviewed articles in English were included. It is possible that unpublished studies or non-peer-reviewed studies in other languages existed. Second, some studies with mixed diagnoses that also included gynecologic cancer had to be excluded because results were not displayed by cancer diagnosis. It is possible that some contributions from patients with gynecologic cancer were discarded. Third, the majority of the studies included in the review were retrospective, so participants’ responses may be affected by recall bias. Finally, the small number of studies included and the premise that studies would not be excluded from the review based on their quality, we need to acknowledge the methodological limitations of some studies, which makes it difficult to draw robust conclusions and, limits generalizability of findings to a wider patients with gynecologic cancer. Studies’ quality was mostly moderate, ranging from 60% to 80%, especially quantitative studies. Small sample sizes, with a considerable number of studies using heterogeneous diagnosis samples with few patients with gynecologic cancer, biased sampling designs and use of measurement tools that lack validation leave room for methodological improvements of future research. In addition, with the exception of Carter et al,^30^ the studies evaluated were retrospective in nature, targeted a variety of outcomes, measured by diverse tools or questions lacking uniformity and were rarely multicentre studies. Despite these limitations, this review provides the first collection of data on this topic for patients with gynecologic cancer. The results have potential to translate to clinical practice and strengthen future research.

To improve clinical practice, this systematic review recommends that a multidisciplinary approach to oncofertility care, integrating psychological support, should be provided to aid patients at the time of diagnosis and throughout survivorship, grounded in a collaboration among different specialities, with clear pathways for timely referral to fertility specialists. Furthermore, the provision of training to the medical team with the necessary communication skills, confidence and knowledge to implement high standard oncofertility care is needed. Supplementing these efforts with the development of educational resources, such as DAs, may increase QOL and patient satisfaction. Additionally, timely identification of patients at risk for mental distress and providing psychosocial interventions is essential to reduce the likelihood of long-term distress.

This systematic review also demonstrated that patients with gynecologic cancer have been much neglected in research regarding the impact of the diagnosis and treatments on their fertility from their own perspectives. Therefore, greater efforts should be made to develop large robust multicenter studies that overcome flaws identified in the existing research, and also provide new knowledge regarding gynecologic patients’ attitudes, perceptions, and decision-making on fertility, fertility preservation options, and alternative family building, taking into consideration cultural, religious, and moral beliefs. Moreover, prospective longitudinal studies are essential to capture possible fluctuations in attitudes and the impact of fertility variables on patients’ mental health over time. Future research should also explore barriers that preclude optimal fertility decision-making to promote a high standard of oncofertility care.

## Conclusion

To the best of our knowledge, this is the first systematic review to assess fertility and parenthood attitudes, perspectives, and decisions of patients with gynecologic cancer. It captures the unique challenges faced by this population regarding treatment impact on reproductive organs and complex fertility decisions, perceptions, and experiences. In reality, a narrow set of patients have the possibility for fertility preservation, since many have advanced disease (often seen in ovarian cancer) and for whom fertility preservation may not be possible. For early stages of disease, the path of pursuing fertility preservation may be also complex. However, it is paramount to consider that for most of the young patients with gynecologic cancer, fertility and parenthood are of high importance, regardless of their clinical circumstances. Therefore, these patients, including the patients for whom fertility preservation is not indicated or requested, should be clinically managed within multidisciplinary oncofertility programs that provide timely, comprehensive, and optimal fertility counseling and support. The continuity of oncofertility care should be available to patients from diagnosis through survivorship.

## Supplementary Material

oyab051_suppl_Supplementary_Tables

## Data Availability

The data underlying this article will be shared on reasonable request to the corresponding author.
