# Supplementary material for: Perspectives of Young Women With Gynecologic Cancers on Fertility and Fertility Preservation: A Systematic Review
Source: Oncologist. 2022 Feb 28;27(3):e251–64. doi: 10.1093/oncolo/oyab051 (PMC8914481; doi:10.1093/oncolo/oyab051)
Supplement: oyab051_suppl_Supplementary_Tables [file oyab051_suppl_supplementary_tables.pdf]

The Oncologist
The official journal of the Society for Translational Oncology

Supplemental Tables for:
Perspectives of young women with gynaecologic cancers on fertility and fertility preservation: A systematic review and metasynthesis
Vânia Gonçalves et al.

Table S1. Quality Assessment MMAT – Quantitative studies

**QUANTITATIVE STUDIES**						
**Study**	Is the sampling strategy relevant to address the research question?	Is the sample representative of the target population?	Are the measurements appropriate?	Is the risk of nonresponse bias low?	Is the statistical analysis appropriate to answer the research question?	**Quality Assessment**
Wenzel et al. (18)	No	Yes	Yes	No	Yes	3 (***), 60%
Wenzel et al. (19)	Yes	Yes	Yes	No	Yes	4 (****), 80%
Carter et al. (20)	Yes	No	Yes	Yes	No	4 (****), 80%
Armuand et al. (21)	Yes	No	Yes	No	Yes	3 (***), 60%
Campos et al. (22)	Yes	Yes	No	No	Yes	3 (***), 60%
Chan et al. (23)	Yes	Yes	Yes	No	Yes	4 (****), 80%
Ameri et al. (24)	Yes	Yes	No	No	Yes	3 (***), 60%
Sobota et al. (25)	No	Yes	Yes	No	Yes	3 (***), 60%
Shah et al. (26)	Yes	Yes	No	No	Yes	3 (***), 60%
Chin et al. (27)	Yes	Yes	No	No	Yes	3 (***), 60%

Table S2. Quality Assessment MMAT – Qualitative studies

**QUALITATIVE STUDIES**						
**Study**	Is the qualitative approach appropriate to answer the research question?	Are the qualitative data collection methods adequate to address the research question?	Are the findings adequately derived from the data?	Is the interpretation of results sufficiently substantiated by data?	Is there coherence between qualitative data sources, collection, analysis and interpretation?	**Quality Assessment**
Komatsu et al. (28)	Yes	Yes	Yes	Yes	Yes	5 (*****), 100%
Mitchell et al. (29)	No	No	Yes	No	Yes	4 (****), 80%

The Oncologist
The official journal of the Society for Translational Oncology

Supplemental Tables for:
Perspectives of young women with gynaecologic cancers on fertility and fertility preservation: A systematic review and metasynthesis
Vânia Gonçalves et al.

Table S3. Quality Assessment MMAT – Mixed Methods studies

**MIXED METHODS STUDY**																
	**QUALITATIVE STUDY**					**QUANTITATIVE STUDY**					**MIXED METHODS STUDIES**					
**Study**	Is the qualitative approach appropriate to answer the research question?	Are the qualitative data collection methods adequate to address the research question?	Are the findings adequately derived from the data?	Is the interpretation of results sufficiently substantiated by data?	Is there coherence between qualitative data sources, collection, analysis and interpretation?	Is the sampling strategy relevant to address the research question?	Is the sample representative of the target population?	Are the measurements appropriate?	Is the risk of nonresponse bias low?	Is the statistical analysis appropriate to answer the research question?	Is there an adequate rationale for using a mixed methods design to address the research question?	Are the different components of the study effectively integrated to answer the research question?	Are the outputs of the integration of qualitative and quantitative components adequately interpreted?	Are divergences and inconsistencies between quantitative and qualitative results adequately addressed?	Do the different components of the study adhere to the quality criteria of each tradition of the methods involved?	**Quality Assessment**
Carter et al. (30)	No	Yes	No	No	Yes	Yes	No	Yes	No	Yes	No	Yes	Yes	Yes	No	2 (**), 40%
